# Circadian preference and quality of life in adults with autism spectrum disorder: a Brazilian cross-sectional self-reported survey

**DOI:** 10.1007/s11136-026-04176-1

**Published:** 2026-03-01

**Authors:** Júlia Grillo Lima, Mônica Thaís S. Macedo, Thais de Oliveira F. Baldo, Marcelo Perim Baldo

**Affiliations:** 1 Centro de Excelência de Pesquisa em Saúde (CEPS), Montes Claros State University - Unimontes, Montes Claros, MG Brazil; 2 Department of Pathophysiology, Cardiovascular Research Center (CPC/LAMICC), Montes Claros State University, Av. Dr. Rui Braga, s/n, Vila Mauriceia, Montes Claros, MG 39401-089 Brazil

**Keywords:** Autism spectrum disorder, Quality of life, Chronotype, Adults

## Abstract

**Purpose:**

Adults with Autism Spectrum Disorder (ASD) often experience significant challenges that affect their quality of life (QoL), including mental health issues, socioeconomic strain, and circadian rhythm disruptions. Chronotype, or an individual’s biological preference for sleep-wake patterns, has been associated with well-being in neurotypical populations but remains underexplored in adults with ASD. This study aimed to investigate the association between chronotype and perceived QoL in Brazilian adults with ASD.

**Methods:**

We conducted a cross-sectional, web-based survey as part of the SOLACE-ASD Brasil project. A total of 439 adults (≥ 18 years) with a self-reported diagnosis of ASD completed standardized questionnaires assessing chronotype (Morningness-Eveningness Questionnaire) and QoL (EUROHIS-QOL 8). Sociodemographic, behavioral, and clinical variables were also collected. Associations were examined using multiple linear regression and ANCOVA, adjusting for potential confounders.

**Results:**

Intermediate chronotype was the most prevalent (40.1%), followed by evening (38.7%) and morning (21.2%) chronotypes. Overall, 60.4% of participants reported low QoL. Evening-type individuals had significantly lower overall and physical health QoL scores than morning and intermediate types. In multivariate analysis, evening chronotype, unemployment, low income, tobacco use, and physical inactivity were independently associated with lower QoL.

**Conclusion:**

Chronotype is an independent predictor of QoL in adults with ASD, with evening preference linked to worse outcomes. These findings highlight the importance of considering circadian biology in public health strategies and suggest that chronotype-targeted interventions could improve QoL in this population.

**Supplementary Information:**

The online version contains supplementary material available at 10.1007/s11136-026-04176-1.

## Abstract (plain language summary)

Adults with autism spectrum disorder (ASD) often face challenges that affect their daily lives, including mental health struggles, sleep problems, and limited access to work or social support. This study explored whether a person’s natural sleep pattern, called chronotype, is related to how they feel about their quality of life. Chronotype refers to whether someone tends to be more active in the morning, in the evening, or somewhere in between. The study included 439 adults with ASD in Brazil who answered online surveys about their daily habits, health, and well-being. Most participants reported low quality of life, especially those who preferred staying up late (evening chronotype). These individuals also had more physical health complaints. The main finding is that having an evening chronotype is linked to lower quality of life in adults with ASD, even when other factors like income, employment, and physical activity are considered. This means that sleep-wake patterns may play an important role in how adults with ASD experience daily life. These results suggest that helping adults with ASD create better sleep routines, or adjusting work and school schedules to fit their natural rhythms, might improve their well-being.

**Supplementary Information:**

The online version contains supplementary material available at 10.1007/s11136-026-04176-1.

## Introduction

Autism Spectrum Disorder (ASD) is a neurodevelopmental condition characterized by persistent difficulties in communication and social interaction, as well as the presence of restricted and repetitive behaviors and/or interests [1]. ASD is inherently heterogeneous, encompassing individuals with a broad range of intellectual abilities, language competencies, and levels of adaptive functioning [1]. While some individuals present with co-occurring intellectual disability, others exhibit average or above-average cognitive abilities, underscoring the importance of conceptualizing ASD as a spectrum rather than a unitary condition. In recent decades, ASD diagnoses have increased substantially worldwide. Current global estimates suggest that approximately 1 in 100 children is affected. In the Americas, prevalence is estimated at around 1 in 160 children; however, reliable prevalence data from low- and middle-income countries remain limited [1, 2]. According to the 2022 Brazilian Demographic Census, approximately 2.4 million individuals reported having received an ASD diagnosis, corresponding to 1.2% of the population aged 2 years and older. Prevalence was higher among men (1.5%) than women (0.9%), with the highest proportion observed in children aged 5–9 years (2.6%). Among adults, ASD prevalence was estimated at 1% [3].

As the prevalence of ASD has increased globally, research and clinical attention directed toward ASD in adulthood have expanded. Although the literature has historically focused on childhood, growing evidence indicates that adults with ASD face substantial challenges related to social inclusion, employment, and mental health, including elevated rates of anxiety, depression, and social isolation [4, 5]. Studies examining quality of life (QoL) in adults with ASD consistently demonstrate significant impairments across multiple domains of well-being [6, 7]. Compared to neurotypical peers, adults with ASD tend to report lower overall QoL, particularly in areas such as social relationships, mental health, employment, and independent living [7, 8]. Psychiatric comorbidities (e.g., anxiety and depression), communication difficulties, and reduced social support are among the factors most consistently associated with poorer QoL in this population [9]. Conversely, interventions that promote autonomy, social participation, and access to specialized and inclusive services have been shown to substantially improve QoL outcomes [10].

In addition to clinical characteristics, several demographic, socioeconomic, and behavioral factors independently influence QoL in adults with ASD. Sex and age differences are consistently reported: women with ASD often experience lower QoL, higher emotional distress, and greater psychiatric comorbidity than men, while younger adults with ASD may report greater uncertainty, perceived stigma, and psychological burden, all of which negatively affect QoL [11, 12]. Socioeconomic conditions, particularly financial hardship, are among the strongest predictors of QoL across populations. Lower income is associated with reduced access to health and support services, limited autonomy, and poorer social and psychological well-being [8]. Health-related behaviors also play a relevant role. Regular physical activity is associated with better mental health, improved stress regulation, and higher QoL among adults with ASD [13], whereas tobacco use is linked to poorer self-rated health, reduced vitality, and lower QoL in the general adult populations and is likely similarly detrimental among adults with ASD [14]. Furthermore, time since diagnosis has emerged as a relevant determinant of QoL. Individuals diagnosed later in life often report prolonged periods of misunderstanding, unmet support needs, and cumulative psychological distress, whereas earlier diagnosis is associated with greater self-awareness, improved access to accommodations, and more favorable QoL outcomes [15].

Beyond these demographic, psychosocial, and clinical factors, increasing attention has been directed towards the role of biological rhythms and sleep-wake patterns in the daily functioning and well-being of adults with ASD. Individual differences in preferred timing of sleep and activity, commonly referred to as chronotype, may contribute to variability in QoL that is not fully explained by traditional determinants. Chronotypes are typically classified as morning, evening, or intermediate types and are regulated by the circadian system, which governs key physiological processes, including sleep, hormone secretion, and metabolic regulation [16]. Conceptually, chronotype is embedded within the broader construct of circadian typology, reflecting stable inter-individual differences in the phase of entrainment of the internal circadian clock relative to the external light-dark cycle [17]. Circadian misalignment, particularly among neurotypical individuals with evening chronotypes, has been associated with an increased risk of sleep disturbances, mood disorders, and cognitive impairments [18, 19]. Importantly, eveningness is not merely a behavioral preference. Evidence from twin studies and large genome-wide association studies indicates that chronotype is partially heritable and reflects biological variation in core circadian clock genes, including PER, CRY, and CLOCK [20]. At the same time, chronotype is shaped by environmental and social factors such as age, light exposure, work or school schedules, and cultural norms, emerging from the dynamic interaction between biological predisposition and social constraints [17].

This interaction is particularly relevant in contemporary societies, where work and school routines tend to impose early start times. Individuals with a delayed biological sleep phase who are required to wake early for social obligations frequently accumulate sleep debt and experience chronic misalignment between internal circadian time and socially imposed schedules, a phenomenon referred to as “social jetlag” [21]. Social jetlag and circadian misalignment are consistently associated with shorter sleep duration, poorer subjective sleep quality, and adverse cardiometabolic and mental health outcomes, particularly among evening types [21, 22]. Thus, evening chronotype may represent a biologically anchored predisposition that becomes disadvantageous when it conflicts with prevailing societal schedules.

Among adults with ASD, emerging evidence suggests a close relationship between circadian disruptions and ASD-related characteristics. Individuals with ASD frequently exhibit atypical sleep patterns, including difficulties initiating or maintaining sleep [23]. These sleep disturbances may exacerbate core and associated features of ASD, such as emotional dysregulation, social difficulties, and psychiatric comorbidities, including anxiety and depression [24]. Moreover, circadian dysregulation may negatively affect daily functioning and overall QoL [18, 25]. Evidence from neurotypical and clinical populations indicates that the link between eveningness and poorer mental health is often mediated or amplified by insufficient or poor-quality sleep, supporting the notion that chronotype and sleep quality constitute intertwined pathways influencing well-being [22].

The relationship between chronotype and QoL has been extensively examined in the general population, with consistent findings indicating that individuals with an evening chronotype report poorer QoL compared with morning or intermediate types [22, 26]. In neurotypical adults, eveningness is associated with a greater prevalence of sleep disorders, chronic fatigue, mood disorders, and impaired social and occupational functioning, all of which negatively affect overall well-being [16, 19]. Among adults with ASD, this relationship may be more pronounced, as circadian disruptions and atypical sleep patterns are common in this population [23]. However, evidence regarding the association between chronotype and QoL in adults with ASD remains scarce. Therefore, this study aimed to examine the association between self-reported chronotype and QoL in a large sample of adults with ASD in Brazil.

## Methods

### Study design and sample characteristics

The present study used data from the SOLACE-ASD Brasil project, a web-based cross-sectional survey. The primary objective of the SOLACE-ASD Brasil project was to investigate the determinants of specific behaviors and mental health conditions among adults with ASD in Brazil. Data collection was conducted through the project’s social media platforms (mainly Instagram^®^ and Facebook^®^), and by the end of the study, the SOLACE Project’s profiles on Instagram^®^ and Facebook^®^ comprised more than 24,000 followers.

Data were collected by disseminating a link to the research questionnaire, which was developed using Google Forms^®^. Participants were recruited through a convenience sampling method via these social media platforms, which were actively maintained by the SOLACE Project. The form was available on the Project’s social media for 90 consecutive days, from June/2021 to September/2021. Participants were allowed to share the link in other virtual environments in a “snowball” fashion. Completing the study form took approximately 25 min and was completely anonymous.

Participants were included if they were aged 18 years or older and self-reported having a definite medical diagnosis of ASD. No independent verification of ASD diagnoses was performed. In Brazil, the diagnostic process for ASD follows internationally established standards, relying on criteria from the Diagnostic and Statistical Manual of Mental Disorders (DSM-5), or the International Classification of Diseases (ICD-10/11) [27, 28]. Clinical diagnosis is typically conducted by trained medical professionals, most commonly child psychiatrists, general psychiatrists, neurologists, or developmental pediatricians, who are legally authorized to diagnose ASD. The diagnostic workflow typically includes a comprehensive clinical interview, a review of developmental history, and direct behavioral observation, with the optional use of standardized instruments.

The first screening question in the Project’s form defined the initial inclusion criterion, in which the respondent confirmed whether they had a definitive medical diagnosis of ASD. If they answered “No,” the form was terminated, and the respondent was not included in the study. No questions were included in the survey regarding the presence of other clinical or psychiatric conditions commonly associated with ASD, such as ADHD, anxiety, or mood disorders. The second screening question concerned the respondent’s age (Are you 18 years old or older? ). If they answered “No,” the form was terminated, and the respondent was not included in the study. Participants were also required to read and digitally sign a consent form presented on the first page of the Project’s form, ensuring informed consent was obtained. Confidentiality was strictly maintained throughout the study, as no personal information was retained, and data were anonymized to protect participant identities. The study received ethical approval from the institutional ethics committee of the Centro Universitário UnifipMoc-Afya (#4.679.268), reflecting adherence to ethical research standards.

Self-report studies are widely used in research with adults with ASD, as they provide direct access to individuals’ perceptions, lived experiences, and subjective outcomes that may not be fully captured by proxy reports. This approach facilitates large-scale data collection, enhances ecological validity, and aligns with contemporary principles of autonomy and neurodiversity-affirming research. Self-report is particularly valuable for evaluating internal states such as QoL, mental health, sleep, and sensory experiences, domains in which adults with ASD are the most accurate informants [12].

At the end of the study period, 444 participants had completed the first section of the online form. However, five participants were excluded as they did not sign the consent form, resulting in a final study sample of 439 valid participants.

### Data integrity procedures

To promote data integrity in this web-based survey, we implemented multiple safeguards before and after data collection. The research form was hosted on Google Forms^®^, which incorporates built-in bot-detection and automated traffic-filtering mechanisms. Before accessing the questionnaire, all participants were required to read and manually agree to an informed consent statement. Recruitment occurred mainly through the official social-media platforms of the SOLACE Project, which maintains an established community of adults with ASD, caregivers, and individuals engaged with ASD-related content. No monetary or material compensation was offered or advertised, reducing the likelihood of fraudulent responses motivated by incentives.

The form incorporated branching logic so that respondents who indicated not having a definitive medical diagnosis of ASD or being younger than 18 years were automatically excluded and could not proceed to the survey. After data collection, we conducted several quality-control checks, including removal of incomplete entries, screening for implausibly short completion times, examination of internal consistency among demographic responses, and evaluation for duplicate or patterned entries suggestive of automated responding. Although Google Forms^®^ does not allow IP-address tracking due to privacy restrictions, to participate in the survey more than once, a different email account would need to be provided. At the end of data collection, no irregular or suspicious response patterns were detected across these verification steps. These procedures collectively aimed to enhance the reliability and authenticity of the final dataset.

### Sociodemographic characteristics

The Project’s form comprised questions about participants’ sociodemographic characteristics, including age, sex, race/skin color, marital status, education level, family monthly income, and employment status. Race/skin color was categorized according to the Brazilian census recommendations into White, Pardos, black, Indigenous, or Asian. Education level was reported as the years the participants attended school/college/university. Employment status was defined as employed, self-employed, unemployed, or retired. Family monthly income was reported based on the number of minimum salaries (268 US dollars) earned by all family members. Additionally, information about health behaviors was also collected, such as tobacco and alcohol use, physical activity engagement, dietary restriction, and use of psychiatric medication. In the survey, “dietary restriction” referred to the self-reported avoidance of specific foods or food groups for personal, sensory, or health-related reasons (e.g., food intolerances, lactose- or gluten-free diets, or selective eating patterns commonly described among adults with ASD).

### Quality of life assessment

To assess participants’ QoL, we used the validated European Health Interview Survey-Quality of Life (EUROHIS-QOL 8) instrument. The EUROHIS-QOL 8 is a generic tool composed of eight items covering various dimensions, including overall QoL, general health, energy, daily living activities, self-esteem, social relationships, finances, and housing. Its application is widely recognized in population studies due to its brevity and ease of understanding [29]. The EUROHIS-QOL 8 has already been translated and validated for use in Brazil [30].

Each item of the EUROHIS-QOL 8 was rated on a 5-point Likert scale, ranging from 1 (“strongly disagree” or “very dissatisfied”) to 5 (“strongly agree” or “very satisfied”). For analysis, the mean of the eight items was calculated to generate a global QoL score ranging from 1 to 5, with higher scores reflecting better perceived QoL [29]. Following previous applications of this instrument in population studies, we classified participants with a mean score ≥ 4 as having high QoL. Importantly, all scores < 4, including neutral mean responses (i.e., a score of 3), were categorized as low QoL, as they represent levels below the threshold typically interpreted as indicating good or satisfactory QoL in this instrument. The reliability of the questionnaire was assessed using Cronbach’s alpha coefficient, which demonstrated satisfactory internal consistency (α = 0.83).

### Chronotype definition

The Morningness-Eveningness Questionnaire (MEQ), developed by Horne and Östberg [31], was used to assess the chronotype of the participants in this study. The MEQ is a widely validated and internationally recognized instrument for classifying individuals into morning, intermediate, or evening chronotypes based on their preferred timing for daily activities such as sleep, work, and leisure. The questionnaire consists of 19 items addressing sleep habits, preferred times for physical and mental activities, and alertness levels at different times of the day [31]. The MEQ has been used in several populations, including adults with ASD [32], and has been translated and validated for use in Brazil [33].

Each item of the MEQ was answered using a specific scoring scale, which varied depending on the question. For example, some questions used 4- or 5-point scales, while others asked participants to indicate specific times for activities like waking up or going to sleep. The total MEQ scores were calculated according to the instrument’s guidelines, ranging from 16 to 86. Participants were classified into three chronotype categories: “morning” (scores ≥ 59), “intermediate” (scores of 42–58), and “evening” (scores ≤ 41) [31]. The reliability of the MEQ was assessed using Cronbach’s alpha coefficient, which demonstrated satisfactory internal consistency (α = 0.88).

### Statistical analysis

All statistical analyses were performed using IBM SPSS Statistics, version 22.0 (IBM Corp., Armonk, NY, USA). Descriptive statistics were used to characterize the sample. Continuous variables were presented as means and standard deviations (SD), while categorical variables were described using absolute and relative frequencies. The normality of continuous variables was assessed using the Kolmogorov-Smirnov test.

Associations between categorical variables were examined using the chi-square test. To investigate the independent contribution of chronotype and other covariates to QoL, multiple linear regression analyses were conducted. The following predictors were simultaneously included in each model: chronotype score, age, sex, monthly family income, physical activity, and presence of comorbidities. Multicollinearity among predictors was evaluated using Variance Inflation Factors (VIF), with values greater than 5 indicating potential collinearity issues. The assumptions of normality, homoscedasticity, and linearity of residuals were verified through visual inspection of residual plots.

Additionally, analysis of covariance (ANCOVA) was employed to compare mean QoL scores and its individual items across chronotype categories (morning, intermediate, evening), while adjusting for potential confounders, including age, sex, family income, physical activity, time since diagnosis, and tobacco use. This approach allowed for the assessment of group differences in QoL items while controlling for relevant covariates.

All statistical tests were two-tailed, and a significance level of *p* < 0.05 was considered statistically significant.

## Results

A total of 439 adults with a self-reported medical diagnosis of ASD participated in the study, with a mean age of 31.3 ± 9.4 years. The mean age was significantly higher among women compared to men (32.5 ± 9.7 vs. 29.5 ± 8.6 years, *p* = 0.001). Of the participants, 61% were female, the majority identified as white (62%), and 59% reported being single. Regarding socioeconomic variables, 74% of the sample reported having completed 12 or more years of education, 70% reported a family income below five minimum wages, and only 31% were employed. The average time since diagnosis was 4.2 ± 6.0 years, significantly longer for men than for women (5.0 ± 6.3 vs. 3.7 ± 5.9 years, *p* = 0.048). Additionally, the mean age at diagnosis was 27.1 ± 10.9 years, significantly younger in men compared to women (24.5 ± 10.4 vs. 28.8 ± 11.0 years, *p* < 0.001).

Table 1 presents the participants’ sociodemographic data, stratified by chronotype. In this sample of adults with ASD, the prevalence of the evening chronotype was 38.7% (*n* = 170), intermediate chronotype was 40.1% (*n* = 176), and morning chronotype was 21.2% (*n* = 93). Regarding behavioral factors, the majority of participants across all chronotypes reported not using tobacco (87.9%) or alcohol (85.7%) and not engaging in regular physical activity (61.7%). However, when analyzing the frequency of dietary restrictions, participants with a morning chronotype reported a higher frequency (53.8%) compared to those with an evening (43.5%) and an intermediate (46%) chronotype. Concerning medication use, 68.3% of participants (*n* = 295) reported regularly taking psychiatric medication. When stratified by chronotype, 71.3% of participants with an evening chronotype reported using psychotropic medication, compared to 67.2% of those with an intermediate chronotype and 64.8% with a morning chronotype (*p* = 0.266).

**Table 1:** Sociodemographic characteristics of adults with ASD stratified by chronotype

	Chronotype					
	Evening (*n* = 170)		Intermediate (*n* = 176)		Morning (*n* = 93)	
	*n*	*%*	*n*	*%*	*n*	*%*
*Sex*						
Women	112	65.9	99	56.2	56	60.2
Men	58	34.1	77	43.8	37	39.8
*Age (years)*						
18–24	55	32.4	47	26.7	27	29.0
25–34	59	34.7	70	39.8	27	29.0
35–44	44	25.9	43	24.4	28	30.1
≥ 45	12	7.1	16	9.1	11	11.8
*Race/Skin color*						
White	113	71.5	103	62.8	58	66.7
Pardos	38	24.1	46	28.0	20	23.0
Black	7	4.4	15	9.1	9	10.3
*Education level*						
≤ 8 years	5	2.9	7	4.0	7	7.6
9–11 years	36	21.2	41	23.3	15	16.3
≥ 12 years	129	75.9	128	72.7	70	76.1
*Marital status*						
Married	56	33.3	51	29.8	42	45.2
Single	101	60.1	112	65.5	46	49.5
Divorced	11	6.5	8	4.7	5	5.4
*Family income*						
< 5 salaries	117	68.8	128	72.7	64	68.8
5–7 salaries	24	14.1	30	17.0	17	18.3
≥ 8 salaries	29	17.1	18	10.2	12	12.9
*Employment status*						
Unemployed	35	34.7	43	42.6	23	22.8
Self-employed	40	44.9	35	39.3	14	15.7
Employed	51	37.2	53	38.7	33	24.1
Student	43	38.9	43	38.9	22	20.4
*Tobacco use*						
No	140	82.8	158	90.3	85	91.4
Yes	29	17.2	17	9.7	8	8.6
*Alcohol consumption*						
No	141	82.9	149	86.6	82	89.1
Yes	29	17.1	23	13.4	10	10.9
*Physical activity*						
No	115	67.6	103	58.5	53	57.0
Yes	55	32.4	73	41.5	40	43.0
*Dietary restrictions*						
No	96	56.5	95	54.0	43	46.2
Yes	74	43.5	81	46.0	50	53.8
*Medication use*						
No	48	28.7	57	32.8	32	35.2
Yes	119	71.3	117	67.2	59	64.8
*Time since diagnosis*						
< 5	133	78.2	123	69.9	75	80.6
5–9	16	9.4	21	11.9	8	8.6
≥ 10	21	12.4	32	18.2	10	10.8

**Fig. 1:**
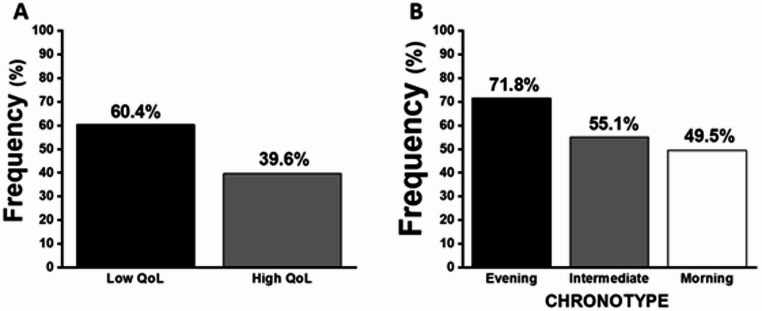
Prevalence of low quality of life according to chronotype among adults with Autism Spectrum Disorder (ASD). **A** Distribution of self-reported low quality of life showing significantly higher prevalence of low QoL in our sample. **B** Frequency of low quality of life stratified by chronotype. Indicating the highest prevalence among individuals with an evening chronotype. Followed by intermediate and morning types

We also investigated perceived QoL in this sample of adults with ASD. Overall, 60.4% of participants reported low QoL (Fig. 1A), with women reporting low QoL significantly more often than men (66.3% vs. 51.2%, *p* < 0.05). When stratified by chronotype, participants with an evening chronotype reported significantly higher frequency of low QoL compared to those with intermediate and morning chronotypes (Fig. 1B). To identify factors independently associated with low QoL, we conducted a multiple linear regression analysis. This model explained 23.2% of the variability in perceived QoL among adults with ASD. Variables that were significantly and independently associated with QoL included chronotype, family income, unemployment, smoking, physical activity, and time since diagnosis (Table 2).

**Table 2:** Multiple linear regression to identify independent variables associated with high QoL in adults with ASD

Model	β	β (SEM)	t	*P* value
*Constant*	19.696		9.964	< 0.001
Chronotypes (*MEQ score*)	0.049	0.114	2.576	0.010
Family income (5–7 salaries)	1.854	0.118	2.611	0.009
Family income (≥ 8 salaries)	3.129	0.188	4.083	< 0.001
Unemployment	−2.568	−0.189	−3.670	< 0.001
Tobacco use	−1.889	−0.109	−2.432	0.015
Practice of physical activity	2.009	0.171	3.911	< 0.001
Time since diagnosis (years)	0.156	0.192	4.387	< 0.001

As shown in Table 3, overall QoL scores were significantly higher among participants with morning and intermediate chronotypes compared to those with an evening chronotype. When QoL was further examined by its specific items, physical health scores were significantly higher among participants with morning and intermediate chronotypes relative to those with an evening chronotype. No significant differences were observed across chronotypes in the remaining QoL items.

**Table 3:** Association between quality of life and its individual items with the chronotype in adults with ASD

	Chronotype			
	Evening (*n* = 170)	Intermediate (*n* = 176)	Morning (*n* = 93)	*P* value
	Mean ± SD	Mean ± SD	Mean ± SD	
Global quality of life	2.797 ± 0.652	2.936 ± 0.663	2.989 ± 0.656	0.045*^#^
How would you rate your quality of life?	3.125 ± 0.873	3.297 ± 0.888	3.339 ± 0.896	0.103
How satisfied are you with your health?	2.836 ± 1.030	2.912 ± 1.034	3.028 ± 1.031	0.359
Do you have enough energy for everyday life?	2.623 ± 0.868	2.800 ± 0.875	2.936 ± 0.877	0.019*
How satisfied are you with your ability to perform your daily living activities?	2.539 ± 1.023	2.747 ± 1.034	2.848 ± 1.041	0.047*
How satisfied are you with yourself?	2.688 ± 1.082	2.674 ± 1.087	2.743 ± 1.089	0.882
How satisfied are you with your personal relationships?	2.689 ± 1.147	2.875 ± 1.154	2.794 ± 1.157	0.330
Have you enough money to meet your needs?	2.695 ± 0.842	2.803 ± 0.849	2.908 ± 0.858	0.146
How satisfied are you with the conditions of your living place?	3.182 ± 1.186	3.382 ± 1.193	3.317 ± 1.195	0.291

The model was adjusted by age. sex. family income. physical activity. time since diagnosis. and tobacco use

* *P* < 0.05 between Evening vs. Morning. # *P* < 0.05 between Evening vs. Intermediate

## Discussion

This study revealed a high prevalence of low QoL among adults with ASD, particularly among women, individuals who are unemployed, physically inactive, or living with a lower family income. Notably, this is one of the first studies to explore the relationship between chronotype and QoL in a large and socioeconomically diverse sample of adults with ASD in Brazil. The results demonstrate that individuals with an evening chronotype report significantly lower QoL scores, especially in the physical health domain, than those with morning or intermediate chronotypes. These findings underscore the importance of considering circadian preferences and social determinants of health when designing public health strategies aimed at improving the well-being of adults with ASD.

Quality of life is a critical outcome for individuals with ASD, particularly in adulthood, when many people face cumulative social, occupational, and health-related challenges [34]. In line with previous studies, the present findings show that adults with ASD report lower QoL scores, especially in the physical and psychological domains. Research has consistently demonstrated that adults with ASD experience poorer QoL compared to neurotypical peers, often due to higher rates of co-occurring conditions such as anxiety, depression, sleep disturbances, and sensory processing difficulties [8, 35]. Furthermore, factors such as unemployment, lack of social support, and reduced access to tailored services significantly contribute to decreased QoL in this population [12].

Importantly, our findings reinforce prior evidence that QoL in adults with ASD is not solely shaped by core autistic traits but is deeply influenced by contextual and behavioral factors. Unemployment was strongly associated with reduced QoL, corroborating international studies that show persistently low employment rates among adults with ASD and the associated psychological burden stemming from social exclusion, financial insecurity, and underutilization of skills [8, 36]. Conversely, engagement in regular physical activity emerged as a robust correlate of higher QoL. This is consistent with systematic reviews demonstrating that exercise can significantly improve sleep, reduce anxiety and depressive symptoms, and enhance self-regulation in adults with ASD [13, 37]. It is important to note that the relationship between chronotype and behavioral factors such as physical activity is bidirectional: while chronotype reflects underlying circadian biology and inherited variation, physical activity can itself influence circadian phase and sleep quality, suggesting a dynamic interaction between predispositions and environmental factors. Socioeconomic status also emerged as a critical determinant of QoL. Higher family income was associated with better QoL outcomes, likely reflecting enhanced access to health care, educational resources, and social services. These results are consistent with ecological models of disability, which emphasize the interplay between individual vulnerabilities and structural inequities [34]. In this regard, efforts to improve the QoL of adults with ASD must address broader social determinants of health, including income inequality and access to culturally competent health services.

Gender disparities also warrant critical attention. Women in our sample not only reported lower QoL scores but were also diagnosed significantly later than men. These findings may reflect a pervasive diagnostic bias that leads to underrecognition and delayed diagnosis of women with ASD, often due to social camouflaging strategies and the inadequacy of male-centric diagnostic criteria [11]. Delayed diagnosis is particularly concerning as it postpones access to targeted interventions and support, potentially exacerbating mental health difficulties and reducing overall well-being [38].

Individuals with ASD exhibit a biological vulnerability to circadian rhythm disruptions, which may intensify the negative effects associated with an evening chronotype. A growing body of evidence indicates that sleep-wake rhythm abnormalities are not merely secondary symptoms but rather core features of ASD neurobiology [39]. Studies have reported decreased nocturnal melatonin secretion, phase-delayed rhythms, and abnormal melatonin synthesis in individuals with ASD, which have been linked to mutations in the ASMT and CYP1A2 genes, both involved in melatonin metabolism [40, 41]. In addition, reduced expression of melatonin receptors and dysfunction in the suprachiasmatic nucleus have been implicated in altered circadian entrainment in individuals with ASD [42]. These abnormalities can cause persistent difficulties in initiating and maintaining sleep, resulting in chronic sleep deprivation, which negatively impacts mood regulation, executive functioning, and sensory processing, domains closely associated with QoL. Importantly, sleep disruption is also predictive of increased caregiver stress and higher rates of comorbid psychiatric disorders [43], further amplifying the functional burden for adults with ASD.

Genetic studies corroborate these findings, identifying associations between ASD and polymorphisms in core circadian clock genes, such as *CLOCK*, *PER1*, *PER2*, *CRY1*, and *NPAS2*, which regulate sleep timing, hormone release, and behavioral arousal [44, 45]. These genetic vulnerabilities may underlie the high prevalence of delayed sleep phase and evening chronotypes among individuals with ASD. In our sample, individuals with an evening chronotype not only reported lower overall QoL but also exhibited poorer physical health QoL, consistent with studies showing that circadian misalignment can lead to metabolic dysfunction, increased inflammatory markers, and impaired immune responses [46, 47].

Beyond these neurobiological mechanisms, behavioral and psychosocial factors likely compound the effects of evening chronotype on QoL. Individuals with ASD frequently exhibit cognitive rigidity, heightened sensitivity to environmental changes, and difficulties with behavioral adaptation, traits that can exacerbate the impact of “social jet lag” [21]. Social jet lag is more pronounced in evening chronotypes and is associated with higher rates of depression, impaired cognitive performance, and emotional dysregulation [48, 49]. In adults with ASD, these effects may be intensified by existing challenges with emotional self-regulation, sensory overload, and anxiety. Indeed, studies have found that adults with ASD with delayed sleep-wake patterns tend to report greater daytime dysfunction, increased social difficulties, and heightened internalizing symptoms, such as anxiety and depression [23, 50]. Another study by Baker and Richdale [23] observed that individuals with ASD who had an evening chronotype had higher levels of insomnia, depressive symptoms, and social withdrawal. These findings support our data showing that evening chronotype is an independent predictor of poorer QoL.

From a translational perspective, the association between eveningness and reduced QoL opens a promising avenue for targeted interventions. Several studies suggest that chronotype is modifiable through behavioral and environmental strategies, such as bright light therapy, melatonin supplementation, sleep hygiene education, and structured activity scheduling [51, 52]. Randomized controlled trials in neurotypical and clinical populations have shown that such interventions can advance sleep phase, reduce sleep latency, and improve mood and daytime functioning, outcomes closely linked to QoL [53, 54]. In ASD populations, preliminary evidence suggests that tailored circadian interventions are feasible and may lead to improvements in sleep continuity, irritability, and adaptive behavior [55, 56]. Given the heightened susceptibility to sleep-related dysfunction in adults with ASD, public health strategies that promote circadian alignment, particularly for those with an evening preference, may yield substantial QoL benefits. Moreover, promoting flexible educational and occupational scheduling could help reduce the burden of social jet lag and facilitate better sleep-health congruence for this population.

The association between chronotype and QoL is also supported by findings from large population-based studies in non-ASD samples. For example, it has been demonstrated that evening chronotypes exhibit significantly poorer self-rated health, lower sleep quality, and more depressive symptoms, even after controlling for confounding variables [57]. Similarly, a population study by Merikanto et al. [22] showed that evening types were over twice as likely to report poor QoL compared to morning types, largely due to social and occupational misalignment. While these findings are compelling on their own, their implications may be even more profound in individuals with ASD, who already contend with social exclusion, stigma, and service inaccessibility. In this context, the association between chronotype and QoL may reflect both biological vulnerability and structural disadvantage.

This study has several limitations that should be considered when interpreting the findings. First, the ASD diagnosis was self-reported, which may introduce information bias and hinder clinical confirmation of cases. Second, the cross-sectional design limits the ability to determine causal relationships between the variables analyzed, such as the impact of chronotype on QoL. Additionally, although the sample was large and socioeconomically diverse, it was predominantly composed of white and female participants, which may limit the generalizability of the findings to other demographic groups. Another limitation is the lack of objective sleep measures or clinical assessment of psychiatric conditions, which could have enriched the analysis of the effects of chronotype on QoL. Finally, self-report surveys may include potential variability in introspective accuracy, difficulties in interpreting abstract questionnaire items among some individuals, and the inability to clinically verify reported diagnoses. Even so, self-report methodologies remain a robust and internationally accepted strategy for population-based studies involving autistic adults, especially when combined with clear inclusion criteria and validated instruments.

The findings of this study highlight the high prevalence of low QoL among adults with ASD in Brazil and underscore the role of chronotype, socioeconomic status, and behavioral factors such as employment, physical activity, and sleep habits in shaping this outcome. In particular, individuals with an evening chronotype reported poorer QoL, suggesting that interventions aimed at circadian rhythm regulation, such as sleep hygiene strategies, chronobiological therapy, or flexible school and work schedules, may be especially beneficial for this group. From a public policy perspective, the results reinforce the need for cross-sectoral approaches that promote employment inclusion, early diagnosis, access to mental health services, and the creation of sensory-accessible and adaptable environments. Considering the social determinants of health, policies that reduce economic inequality and expand the availability of personalized and culturally competent services are essential to promote well-being and equity among adults with ASD.

## Supplementary Information

Below is the link to the electronic supplementary material.

Supplementary Material 1

## Data Availability

Additional data are available from the corresponding author on reasonable request.
